# Protocol for an evaluation of the Designing Communities to Support Healthy Living in Aging Residents Study

**DOI:** 10.1186/s13690-021-00691-4

**Published:** 2021-10-07

**Authors:** Jodie A. Stearns, Hui Ren, John C. Spence, Hayford Avedzi, Karen K. Lee

**Affiliations:** 1 Division of Preventive Medicine, Department of Medicine, Faculty of Medicine and Dentistry, University of Alberta, Edmonton, Alberta Canada; 2 Faculty of Kinesiology, Sport, and Recreation, University of Alberta, Edmonton, Alberta Canada; 3 Housing for Health, Division of Preventive Medicine, Faculty of Medicine and Dentistry, University of Alberta, 1902 College Plaza, 8215 112 St NW, AB T6G 2C8 Edmonton, Canada

**Keywords:** quasi-experiment, built environment, food environment, older adults, physical activity, healthy eating, social connections, qualitative, environmental audits

## Abstract

**Background:**

In collaboration with building developers, the Housing for Health team is contributing to the design of community-based congregate living facilities to support healthy living in older adults. There may also be opportunities to improve the surrounding neighbourhoods by collaborating with the municipalities where the developments are located. We will evaluate whether one or more of these comprehensive interventions lead to changes in the perceived, microscale, and macroscale neighbourhood-built environment (BE) and amenities, and impacts on the physical activity (PA), healthy eating, and social connections of residents. In parallel, we will gather qualitative data to provide a more in-depth understanding of how the BE may facilitate or hinder resident’s healthy living outcomes.

**Methods:**

This project employs a quasi-experimental pre-post design with at least one or more intervention and control sites. The quantitative BE evaluation will include pre- and post-intervention assessments of neighbourhood macroscale (e.g., layout of communities) and microscale (e.g., street details and characteristics) changes using Geographical Information Systems (GIS) and Microscale Audit Pedestrian Streetscapes (MAPS) audits, respectively. The quantitative resident evaluation will include self-report (i.e., surveys) and objective assessments (i.e., accelerometers, Global Positioning System [GPS]) of residents at baseline (3-6-months pre-move-in) and follow-up (3-6-months and 9-12-months post-move-in if possible). The qualitative resident-environment component will involve in-depth semi-structured interviews post-intervention with building residents, family members, and stakeholders involved in the design/development and/or operation of the intervention site(s). Participant observations will be completed in the building and neighbourhood environments of the intervention site(s).

**Discussion:**

Findings will provide evidence on whether and how comprehensive changes to the BE and amenities of at least one congregate living facility and the surrounding neighbourhood can impact PA, healthy eating, and social connections of older adults. Successful intervention elements will be scaled up in future work. We will disseminate findings to a broad audience including the scientific community via peer-reviewed publications, conference presentations, and discussion panels; and the private, public, and not-for-profit sectors via reports, public presentations, and/or communications via our partners and their networks.

**Trial registration:**

Protocol ID: 1819-HQ-000051. ClinicalTrials.gov ID: NCT05031273. Registered 29 June 2021 with ClinicalTrials.gov.

## Background

Engaging in regular physical activity (PA), eating healthily, and being socially connected contribute to quality of life, and prevention and management of disease throughout the older years [1–3]. Engaging in aerobic PA of moderate or higher intensity also helps older adults maintain their functional independence longer [4]. Unfortunately, many older adults are insufficiently active. Only 17% of Canadian adults aged 60–79 years meet recommended guidelines, as determined by accelerometers [5]. Rates of social contact also substantially decrease in older age [6], and many do not consume sufficient amounts of fruits and vegetables [2]. Additionally, as the proportion of the global population aged 60 years and older is expected to double to 2.1 billion by 2050 [7], associated health care costs are of concern. Thus, public health interventions designed to support healthy living in older adults are imperative, to ensure both quality of life for growing older populations, along with reducing costs of healthcare [8].

Older adults are important contributors to our communities; yet they require additional supports to help deal with inevitable physical and social changes that occur with aging [9]. In response to global aging, the World Health Organization (2007) is promoting the creation of “age-friendly communities”, which includes designing housing and physical environments to meet needs in people of all ages, supporting the wellbeing of older residents, and ensuring cities are thriving. As many older adults are retired and tend to spend more time within their home and neighbourhood, the design of communities may be particularly important for this population [10].

According to the ecological model of age-friendly communities [11], a constellation of physical and social factors in the interpersonal, community, and policy environments interact to either support or hinder health in individuals. Consistent with this model, a recent review of the literature concluded that neighbourhoods that promote overall PA in older adults are those that are safe and walkable, with aesthetically pleasing scenery and access to destinations and services [12]. Different features however may be important for different types of physical activities. Kerr, Rosenberg [10] proposed that access to destinations, connectivity of streets, and access to transit are likely the most important factors for transportation walking, whereas safety, aesthetics, and parks are more important for recreational walking. As many older adults live in congregate living developments, it is also important to understand how features of these buildings and surrounding sites can support or hinder the healthy living of residents [13]; yet much of the research to date has focused on the outdoor neighbourhood built environment (BE).

Given the decline in mobility and physical function associated with aging, accessibility to healthy food options (e.g. fresh fruits and vegetables) within a walking distance is crucial for healthy aging [14]. Diet quality among urban dwelling older adults has been shown to be associated with the proportion of fast food outlets and stores selling healthy foods [15]. BE elements can also enhance social connections in older adults with ramps to access buildings linked to perceived social connectedness [16].

Although evidence is accumulating on the importance of the BE for healthy living in older adults, much of the research is cross-sectional, and does not allow for establishment of temporal precedence [17]. Many also do not rule out alternative causal explanations (e.g., residential self-selection). To provide stronger evidence for the importance of the BE for healthy living, researchers have started to evaluate impacts of BE interventions on health outcomes [18, 19]. Such designs are vital as they allow us to isolate the effect of the BE on healthy living, and allow for conclusions around causality [17]. Because randomized control trials are difficult to implement in real-world community settings, quasi- and natural-experiments are important research designs in public health [20]. To date, short-term urban and landscape street design interventions have resulted in changes to perceptions of walkability and street safety, and perceptions of being more active post-intervention, with no change in social engagement or outdoor activity [21, 22]. An urban greenspace intervention however did not impact well-being behaviours and use of the spaces by older adults [23]. Given that the environment includes many features, multiple co-occurring interventions may be necessary to produce meaningful impacts on behaviour [24]. Interventions may also be more effective if they are designed based on consistent correlates identified in the literature [24].

Gathering information on intermediate variables can help provide understanding around the pathways between the BE and health outcomes [17]. The Capability, Opportunity, Motivation, and Behaviour (COM-B) model represents a behavioural system of factors (i.e., beliefs) that interact and reciprocally influence health-related behaviours [25]. The antecedents of behaviour include perceptions of psychological (e.g., ability to reason and comprehend) and physical capability (e.g., physical strength and skill), social (e.g., cultural and linguistic factors) and physical opportunity (e.g., BE), and motivation which includes reflective (e.g., conscious decisions) and automatic processes (e.g., habits, emotional reactions). This behavioural system is linked to intervention functions and policy categories via the behaviour change wheel, with “environmental restructuring” being the critical function for our study.

The Designing Communities to Support Healthy Living in Aging Residents Study is designed to address several of the aforementioned gaps in the literature. This study is a part of the Housing for Health project, which is a five-year initiative (2018–2023) in Alberta, Canada that investigates how the BE of housing developments and neighbourhoods supports health and protects against disease. In collaboration with building developers, the Housing for Health team is contributing to the building and site design of community-based congregate-living facilities to support healthy living in older adults. The team is also meeting with the municipalities where the developments are located to explore opportunities to improve the surrounding neighbourhoods. To evaluate the impacts of one or more of these comprehensive interventions, we will audit the microscale and macroscale BE to document implemented design and amenity elements, along with changes that occur due to factors outside of the study. We will then examine whether and how these design and amenity elements impact the residents’ healthy living outcomes and perceptions of neighbourhood built and food environments. In parallel, we will gather in-depth understandings of how the BE may facilitate or hinder resident’s healthy living behaviours using qualitative methods.

The quantitative environment evaluation will examine the following objectives.

1. To assess whether microscale and macroscale neighbourhood BE features and amenities supporting PA, healthy food consumption, and social connections at the intervention site(s) descriptively appear to have changed more than control site(s).
2. To assess whether macroscale neighbourhood BE features and amenities impacting PA, healthy food consumption, and social connections are more supportive in the intervention site neighbourhood(s) post-intervention compared to the residents’ old neighbourhoods.

The quantitative resident evaluation will examine the following objectives.

3) To assess whether residents from the intervention site(s) have greater improvements in PA, healthy food consumption, and social connections, compared to residents from control site(s).
4) To assess whether perceived built and food environments of residents improve more in the intervention site(s) compared to control site(s).
5) To assess whether change in capability, opportunity, and/or motivation mediate the effect of the intervention on changes in PA, healthy food consumption, and social connections.

The qualitative resident-environment component will explore the following objectives.

6) To document detailed features of the BE and people-BE interactions at the intervention site.
7) To gather insights on how the BE at the intervention site impacts residents’ PA, healthy food consumption, and social connections through capability, opportunity, and motivation.

## Methods/design

### Study design

The quantitative component will follow a quasi-experimental pre-post design as the assignment to groups is non-random and our research team is working with developers and municipalities to influence the interventions. However, uptake of suggested building and site scale recommendations is ultimately up to the discretion of the developers and architects, and uptake of neighbourhood-scale recommendations is ultimately up to the discretion of the municipalities.

### Setting and residents

The evaluation will include at least one intervention site and one or more control site (we are aiming for two or more) situated in Edmonton and nearby towns in northern Alberta, Canada. This region experiences long, cold, and icy winters, and mild to hot summers.

The intervention site(s) will be planned upcoming community-based congregate living building(s) where our team has the potential to influence design and amenities of building(s), site(s), and/or neighbourhood(s), and where an evaluation of the impacts of moving into the new building(s) on resident’s healthy living outcomes is possible during the timeframe of the grant (October 2018 to October 2023). The units may be rental or life lease units. We plan to select standard community-based congregate seniors housing with few health-promoting features as control site(s). The intervention and control sites will be as closely matched as possible on city (e.g., size), building (e.g., building type, number of stories, number of independent living units), and resident demographics (e.g., age, income). The timelines for construction are controlled by the developer(s), and dependent on many external factors (e.g., approvals from the municipality, grant funding or loan approvals, COVID-19-related factors, availability and cost of building materials and supplies). Therefore, exact locations of the intervention and control site(s) are still being finalized to ensure we can complete the evaluation research within grant timeframes. Several delays have already occurred.

### Intervention

Based on evidence of BE features that are associated with older adults’ PA, healthy eating, and social connections, as well as preliminary data collected by our team, design recommendations for improving health-promoting features of buildings, sites, and local neighbourhoods are being made. We are working closely with building developers and architecture teams to integrate intervention elements at the building and site scales. We also plan to work with municipalities to integrate intervention elements at the neighbourhood scale where possible. When developers have specifically chosen upcoming sites situated within neighbourhoods with amenities close by, the intervention(s) will also include the existing healthy-features of the neighbourhood(s) (e.g., mixed-use).

Design intervention elements will be tailored to the intervention site(s). The main evidence-based health-promoting features recommended include: more visible stairwells through strategies like open stairwells or stairwells with glazed doors and walls [26]; improvements to stairwell aesthetic and safety designs [27]; indoor and outdoor PA spaces such as multigenerational play and exercise spaces [28]; prompting signage for spaces such as stairs and other amenities [29]; patio/balcony included for each unit with visibility of the patios of other units (based on preliminary research by our team); continuous sidewalks or multi-use paths that connect the building to neighbourhood amenities with benches for resting, pedestrian-scale lighting, and sidewalk handrails or heating [30]; gardening spaces such as greenhouse and/or raised garden beds [31]; bicycle infrastructure such as shared bicycles/adult tricycles, bicycle lanes, and bicycle parking [32]; additional healthy commercial amenities onsite or in neighbourhood [33]; and farmers markets onsite or in neighbourhood [34].

### Stakeholder involvement

The quantitative portion of this project has been guided by a research and evaluation advisory group consisting of members from different sectors including government, private, community, not-for-profit, and multiple academic faculties. This group has advised on research design and selection of measures and will continue to provide advice throughout the different phases of the research such as dissemination.

### Quantitative environment evaluation

#### Procedures

Microscale and macroscale audits will be conducted at the intervention and control site(s) pre- and post-intervention. Macroscale audits will also be completed in the residents’ neighbourhoods before their relocation to the intervention site.

#### Measures

***Microscale audits***: The Microscale Audit of Pedestrian Screetscapes (MAPS) - Full will be used to assess the microscale neighbourhood BE of the sites [35]. This audit tool is designed to approximate a person’s typical walking experience, and usually involves in-person environment audits. Consistent with the MAPS manual, our team will audit all 400-meter street network routes to relevant destinations, starting on the street closest to the development’s main entrance and taking the shortest route to the destinations [35]. Relevant destinations include those that are important for PA (e.g., parks), healthy food access (e.g., grocery stores), and social connections (e.g., libraries), and those that are common destinations for older adults, as determined by other Canadian studies [36, 37] and some pilot work by our team. In addition, because older adults also engage in walking for leisure purposes [38], all additional 400-meter street-network routes around the housing sites not leading to a relevant destination will be assessed. There are four sections in the MAPS tool: routes, crossings, street segments, and cul-de-sacs. Routes items (25 items) examine features related to land use and destinations, transit stops, street amenities, traffic, hardscape and softscape aesthetics, and the social environment. Crossings items (12 items) examine features at every intersection or crossing on the route, including crosswalks, slopes, crossing widths, crossing signals, and pedestrian protection. Street segment items (28 items) examine segment features, including sidewalks, street buffers, sidewalk slope, bicycle facilities, shortcuts, visibility from buildings, building heights, setbacks, and trees. Lastly, cul-de-sac items (12 items) are collected only when one or more cul-de-sacs are present within 123-meters of the development, and include size and condition of the surface area, slope, and amenities [35]. Scoring for MAPS-Full provides an overall route score along with subsection scores for destinations and land use; streetscapes; and aesthetics and social. MAPS items and subscales have been shown to have moderate to excellent reliability [39].

In addition to existing items in the MAPS-Full tool, several audit questions were added to reflect our population (e.g., number and features of benches, washroom/restrooms, buildings with visually-appealing features), winter-city context (e.g., ice and snow, skating rink), socially-connecting features (e.g., outdoor dining areas; house porches; multifamily residential buildings balconies that allow neighbours to interact), and COVID-19-related features (e.g., width of sidewalk $\ge$ 2 m), and other features deemed important by the team (e.g., wayfinding signage, sidewalk material, pedestrian overpass/underpass/bridge, tactile surface indicator, bicycle parking, transport options, sidewalk materials, ramps to access buildings). These questions will be scored separately from the original MAPS items.

Staff training on the MAPS tool include a presentation and discussion of the protocol, in-person practice routes with a certified rater, and the independent rating of five routes. To become a certified rater, staff must achieve an agreement of ≥ 95 % with a certified rater for at least four of the five independently rated routes [39]. Additionally, 10 % of routes at each site will be completed by a second rater to ensure consistency of audits.

***Macroscale audits***: Several macroscale BE features will be assessed using Geographical Information Systems (GIS). Data sources will vary by variable, and will likely include DMTI, OpenStreetMaps, Google Maps Hybrid, and city-specific open data. Considering amenities are changing rapidly during the COVID-19 pandemic, we will compare destinations through all sources and discrepancies will be investigated (e.g., calling a business to see if they have closed). GIS variables will include both composite (e.g., walkability score for the neighbourhood) and specific measures (e.g., access to parks and health care services).

#### Analyses

Microscale and macroscale neighbourhood environment changes pre- to post-intervention will be compared descriptively between the intervention and control site(s). Intervention site(s) macroscale neighbourhood environment post-intervention will also be compared descriptively to the residents’ old neighbourhoods.

### Quantitative resident evaluation

#### Participants

Participants will be recruited with assistance from each facility’s manager (e.g., hosting an information session where residents sign up for a time and day to complete data collection, posters hung on the walls and placed under residents’ doors). Men and women aged 18 and older and living in an independent living unit in an intervention or control site will be invited to participate. We expect most participants to be older adults aged 65 and older. Based on pilot data collected by our team, we expect a 50–55 % participation rate.

#### Procedures

Measurements will occur before and after changes to the BE and amenities are introduced. Residents moving into the new development(s), and current residents at the control site(s) will participate in one baseline measurement (3-6-months before move-in) along with two follow-up measurements if possible (3-6-months and 9-12-months post-move-in).

The researchers will visit the participants at the development where they live for a one to two hour scheduled appointment. First, researchers will describe the study and obtain written informed consent from residents regarding their willingness to participate. Second, participants will complete a survey on their health and health behaviours, perceptions of their neighbourhood environment, and socio-demographic characteristics. Third, their height and weight will be measured using a standiometer and scale. Fourth, they will be asked to wear an accelerometer and Global Positioning System (GPS) device on a waistband for seven consecutive days during all waking hours and to complete a logbook and travel diary. They will be trained on how to position the accelerometer on their right hip and the GPS on their left hip. Participants will receive a $20 gift card for participating in all parts of the study.

#### Measures

***Physical activity***: Objective measures of PA will be obtained using Actigraph wGT3X-BT accelerometers (Actigraph LLC, Pensacola, FL). Decision rules will follow recommendations of Migueles, Cadenas-Sanchez [40] for use with Actigraph accelerometers. Data will be collected in 60 s epochs with a sampling frequency of 60 Hz and the normal filter. Non-wear-time will follow the Choi, Liu [41] algorithm. Days of PA measurement will be considered “valid” if the device is worn for 10 or more hours per day. Four or more days of wear is considered a “valid” measurement week. The main outcomes of interest are average steps per day, minutes of total PA per day, and minutes of moderate-to-vigorous PA (MVPA) per day.

Participants’ location will be tracked using Qstarz BT-Q1000XT Travel Recorder XT GPS devices. These are satellite-based global navigation systems that measure participants’ location every five seconds. A “valid” measurement week will be considered four or more days of wear. Exact GPS variables are still being finalized but will likely include transport and leisure walking. We plan to use the HABITUS software [42] to merge and process accelerometer and GPS data.

To supplement GPS and accelerometer measurements, participants will also complete a travel diary and logbook. They will record their wake and bedtimes, and times when the devices are taken off, and details on where they travel.

Self-report measures of leisure and transport PA from the past seven days within the neighbourhood will be measured using the Neighbourhood – International Physical Activity Questionnaire (N-IPAQ)[43]. Self-reported stair climbing will be assessed with the question “How many total floors of stairs throughout the day do you walk UP on an average weekday at your CURRENT home building?” (responses: 0 floors, 1–2 floors, 3–6 floors, 6 or more floors, unable to walk up the stairs).

***Dietary assessment***: Frequency of fruits and vegetable, salty food, and sugar-sweetened beverage consumption will be assessed using a food frequency questionnaire adapted from the Nutritional Environment Measures Survey – Perceived Nutrition Environment (NEMS-P)[44].

***Social connections***: Connections to people in one’s neighbourhood will be captured using survey questions of sense of belonging [45], community satisfaction [46], local social ties [47], and social cohesion and trust [48].

***Capability, Opportunity, and Motivation***: Physical opportunity will be assessed as perceptions of healthy food access in the neighbourhood using the NEMS-P [44]. Items will assess access to stores and restaurants, store availability of healthy food choices, store prices of fruits and vegetables, store placement of healthy items and nutrition information, availability of healthy options at restaurants, restaurant promotion of healthy options and nutrition information, and costs of healthy options at restaurants.

Physical opportunity will be assessed as perceptions of neighbourhood supports for PA using the Physical Activity Neighbourhood Environment Scale (PANES)[49]. This scale captures residential density, land mix use, street connectivity, proximity to neighbourhood recreation facilities, pedestrian infrastructure, bicycling infrastructure, aesthetic qualities, social cues for PA, traffic and crime safety, and access to a working automobile.

Physical capability around PA will be assessed using two questions from the short form health survey (SF-12)[50]. Two questions on functional limitations ask “Does your health now limit you in these activities? If so, how much?” Activities include (a) moderate activities such as moving a table, pushing a vacuum cleaner, bowling, or playing golf, and (b) climbing several flights of stairs.

Questions on motivation, capability, and social opportunity may be assessed using generic items designed specifically for the COM-B [51]. Specific outcomes included in these questions will be selected based on the final design and amenity intervention elements that are implemented at the site(s). For example, if healthy stairwell design elements are implemented, then stair climbing will likely be included as an outcome.

***Body mass index (BMI)***: Measurements of height and weight will be taken using the Canadian Physical Activity, Fitness and Lifestyle Appraisal procedures [52]. Specifically, body weight in Kilograms (nearest 0.1 kg) and height in meters (nearest 1.0 mm) will be measured twice in each participant with light clothing and no shoes using a portable physician-grade stadiometer and scale (Health o Meter). BMI will be calculated as kg/m^2^.

***Socio-demographic information***: Age, gender, ethnicity, physical and mental health status, marital status, smoking and alcohol use, employment status, income, education, and falls in the past year, will be measured using standard questionnaire items.

#### Analyses

Differences in baseline characteristics (e.g., demographic factors and outcome measures) of residents in the intervention and control site(s) will be investigated using independent samples t-tests. Any differences identified may be considered as covariates in the main analyses. We will attempt to control for any unusual events and socio-economic or political influences that occurred during the study period. Covariates may include age, gender, building site, functional ability, car ownership, socio-economic status or other demographic variables that differ between residents of intervention and control sites at baseline.

We intend to run a difference-in-difference analysis to test for differences between intervention and control groups in change from baseline to 3-6-month post-move-in, and change from baseline to 9-12-month post-move-in. The type of analysis will depend on the measurement unit of each outcome (e.g., linear regression for continuous outcome variables, logistic regression for categorical outcome variables). For the primary outcome of PA, a difference-in-difference linear regression analysis with a continuous outcome will have 80 % power with 107 participants for a model with five main predictor variables and three covariates at α = 0.05 [53].

Depending on how many units the final intervention and control sites have, data may be collected on couples and a multi-level modeling approach used to control for clustering within unit. The approach that is best able to maximize power will be chosen.

### Qualitative resident–environment component

#### Methods

Post-move-in we will conduct in-depth interviews with residents, family members, and stakeholders at the intervention site(s), along with participant observations. A semi-structured interview guide will be used during the interviews while also allowing participants to tell relevant stories [54]. Participant observations will include researcher observations and interactions with people in the environment, which will allow us to better understand people-BE interactions [55].

#### Participants

Interview participants include residents living in the intervention site(s) and their family members, along with stakeholders involved in the design and operation of the site(s). Stakeholder participants include but are not limited to building developers, architects, landscape architects, neighbourhood planners, building operators and staff members, and community commercial amenity owners. For participant observations, people present in the BE will be observed, such as building residents, visitors, building staff, and people living or working in the surrounding neighbourhood(s).

#### Procedures

***In-depth interviews***: Building residents living in the intervention site(s) will receive study recruitment materials from the building operator, and residents can share these materials with family members. Interested residents and/or their family members will contact the researcher to set up interview(s). Researchers will invite stakeholders to participate in individual interviews, either through personal connections with the operator or through informal introductions at the site. Interviews will be semi-structured and take 1-1.5 h. The interview guide will include questions about the lived experiences (building residents and family members) or work experiences (stakeholders) of residents’ PA, healthy eating, and social connections in the building, site, and neighbourhood environments. Questions on residents’ capability, opportunity, and motivation for being physically active, eating healthy, and staying socially connected will also be explored. Participants will be encouraged to share other stories. Interviews will be conducted in-person or virtual, depending on the participants’ preference and policies relevant to the COVID-19 pandemic. We will use audio recorders to record the interviews. During the interviews we will take photos (in-person interviews) or screen shots (virtual interviews) of BE features referred to, along with notes and sketches. Reflections will be undertaken immediately after interviews.

***Participant observations***: We will complete in-person observations in the building(s), site(s), and neighbourhood(s) during the daytime and nighttime in different seasons at the intervention site(s). We will observe physical spaces and amenities used by people, and how people’s use of these spaces and amenities promotes PA, healthy eating, and social connections. During observations, we will approach people in the environment to discuss their experiences of using the spaces and amenities. We will also observe detailed features of different design and amenity intervention elements implemented in the building(s), site(s), and neighbourhood(s). Collected data will include photographs, field notes, and sketches. Reflections will be completed immediately after data collection.

#### Data analyses

Interview recordings will be transcribed verbatim. Interview transcripts, photographs/screen shots, field notes and sketches, and reflective notes will be analyzed using thematic content analysis [56]. Researchers will code data by recurrent themes. Themes will include the capability, opportunity, and motivation to be physically active, eat healthily, and stay socially connected. However, new domains including themes that do not fit with the pre-defined domains may also be developed.

#### Research rigor

Several strategies for qualitative research rigor will be employed, including triangulation through multiple data collection methods, the researchers’ prolonged engagement with the site during data collection, and journal-keeping through reflection notes both during and immediately after data collection [56].

### Ethics approval

The Research Ethics Board at the University of Alberta has approved the research involving people (Pro00092947, Pro00094863). All interview and quantitative resident evaluation participants will provide written informed consent. During COVID-19 restrictions, ethics currently only allows virtual interviews, and outdoor site observations without communications with people. The research team will submit an amendment for in-person interviews and participant observations of indoor and outdoor spaces when restrictions are lifted. The macroscale and microscale environmental assessments of the public realm do not require ethics approval.

## Discussion

This project will provide valuable information on whether partnering with developers, site design teams, and municipal officials can result in comprehensive and meaningful changes to the perceived, microscale, and macroscale BE of at least one community-based congregate living facility and the surrounding neighbourhood. Additionally, it will show whether comprehensive changes to the BE can have an impact on the healthy living outcomes of older adults. Quantitative and qualitative findings are expected to be used by practitioners such as architects, landscape architects, planners, property developers, and municipalities to understand how the BE can be improved to support older residents’ healthy activities in future projects and policies. In the future, our team plans to scale up this work to similar settings, as well as to expand this work to other populations (e.g., populations with other socio-economic circumstances). Findings will be disseminated to a broad audience including the scientific community, private, public, and not-for-profit sectors, and community residents via peer-reviewed publications, non-academic reports, academic and practitioner conference presentations and discussion panels, public presentations, news media interviews, and communications with our partners and their networks. The Housing for Health team currently has over 200 partners from multiple sectors in Alberta and across Canada, including academics, developers, architects, urban planners and designers, city officials, and community groups.

To gather greater evidence on the causal impacts of the BE on healthy living, it is now recommended that researchers in this area focus on research designs that support causal inferences, such as quasi-experimental studies [24]. Therefore, the quasi-experimental pre-post design is a key strength. The comprehensive intervention, including potential improvements at the building, site, and/or neighbourhood scales, is a benefit as many BE interventions are focused on small scale changes (e.g., to the street design), which may not be sufficient to change behaviour. The use of theory to guide this work is also an important strength as it has allowed us to identify key intervention mechanisms to test. Use of both objective and self-report measures of PA is a strength as accelerometers and GPS devices provide data that does not rely on participants’ memory recall, whereas self-reported PA can capture PA in different settings (e.g., exercise class, gym attendance). We will also measure total PA which captures PA required for optimal health benefits, along with domain-specific PA which can provide information on the types of PA influenced by the intervention. Behavioural specificity of outcomes is important as different features of the environment may increase some types of PA but not others [10]. Objective measurement of the BE and food environments using macroscale and microscale assessments along with survey measurements is also a strength as different people perceive the BE in different ways, and these perceptions will not always be consistent with objective assessments [57]. Finally, complementary qualitative work is an important strength as it will provide a more in-depth understanding of the factors that influence residents PA, healthy eating, and social connections within the BE.

The study has limitations that should be mentioned. As it will be conducted in Alberta, Canada and may only include one intervention site with participants of a particular socio-economic status, findings may not generalize to older adults in other socio-economic situations or living in other regions. Although we will work for saturation and depth of data from qualitative interviews, we expect the number of interview participants to be relatively small. Therefore, we will not be able to capture the lived experiences of all residents.

The Housing for Health website [58] provides an overview of the entire project that the study is a part of.

## Data Availability

Not applicable.

## Abbreviations

BE: built environment
COM-B: Capability, Opportunity, Motivation, and Behaviour (COM-B)
GIS: Geographical Information Systems
GPS: Global Positioning System
MAPS: Microscale Audit of Pedestrian Streetscapes
MVPA: Moderate-to-vigorous physical activity
NEMS-P: Nutritional Environment Measures Survey – Perceived Nutrition Environment
PANES: Physical Activity Neighborhood Environment Scale
N-IPAQ: Neighbourhood – International Physical Activity Questionnaire
PA: Physical activity
